# Supplementary material for: Mechanical Loading Induces Distinct and Shared Responses in Endothelial and Muscle Cells and Reveals Exercise‐Like Molecular Profiles
Source: FASEB J. 2026 Jul 13;40(14):e72132. doi: 10.1096/fj.202503958RRR (PMC13361157; doi:10.1096/fj.202503958RRR)
Supplement: Supplementary file 1 — Figure S1: Effects of mechanical loading on cell integrity, cell membrane damage, cell viability and orientation of endothelial cells. (A) Total RNA yield was similar between the nonloaded and loaded cells in all cell types, indicating that no significant detachment of cells occurred (n = 6). (B) Mechanical loading did not significantly affect the extracellular lactate dehydrogenase (LDH) activity, a marker of cell membrane damage and cell death (n = 4). LDH activity was not determined from the media of HCMECs because the media were not collected from those experiments. (C) Representative light‐microscope images of HUVEC and Human skeletal muscle (HSkM) cells before and after mechanical loading. (D) Histograms showing the orientation of HUVEC cells in nonloaded and loaded conditions (n=3, depicted in different shades) around the x‐axis (perpendicular to the direction of stretch). (E) Difference in the fraction of cells oriented perpendicular to the direction of stretch (±30° around the x‐axis) between conditions. *p < 0.05 (Student's t‐test). Figure S2: Gene set enrichment of GO:BP terms from overrepresentation analysis with ShinyGO web‐application. Analysis was done by comparing significantly upregulated or downregulated genes to background (all expressed genes in samples after filtering). (A) HUVEC top 10 enriched pathways from upregulated genes. (B) HUVEC top 10 enriched pathways from downregulated genes. (C) HCMEC top 10 enriched pathways from downregulated genes. (D) HSkM top 10 enriched pathways from upregulated genes. No significantly enriched (FDR ≤ 0.05) GO:BP pathways were found from upregulated HCMEC genes or downregulated HSkM genes. Results were visualized by the ShinyGO web‐application. Figure S3: Induction of genes of the Endothelial cell development pathway (GO:0001885) in response to mechanical loading in HUVECs and acute aerobic exercise in human skeletal muscle (HSkM) tissue. (A) A Venn diagram of the induced genes. (B) A Venn diagram of significa [file FSB2-40-e72132-s004.pdf]

Supplementary Figures of the article:

Mechanical loading induces distinct and shared responses in endothelial and muscle cells and reveals exercise-like molecular profiles

Sakari Mäntyselkä[1*], Erik Niemi[1*], Laura Ylä-Outinen[1], Kalle Kolari[1], Liina Uusitalo-Kylmälä[1], Alfredo Ortega-Alonso[1], Roosa-Maria Liimatainen[2], Vasco Fachada[1], Perttu Permi[2,3,4], Elina Kalenius[2], Juha J Hulmi[1#], Riikka Kivelä[1,5,6#]

[1]Faculty of Sport and Health Sciences, NeuroMuscular Research Center, University of Jyväskylä, Jyväskylä, Finland
[2]Department of Chemistry, University of Jyväskylä, Jyväskylä, Finland
[3]Department of Biological and Environmental Science, University of Jyväskylä, Jyväskylä, Finland
[4]Institute of Biotechnology, Helsinki Institute of Life Science, University of Helsinki, Helsinki, Finland
[5]Stem Cells and Metabolism Research Program, Research Programs Unit, Faculty of Medicine, University of Helsinki, Helsinki, Finland
[8]Wihuri Research Institute, Helsinki, Finland

* Equal contribution as first authors

# Equal contribution as senior authors

Correspondence to Riikka Kivelä (riikka.m.kivela@jyu.fi) & Juha J Hulmi (juha.hulmi@jyu.fi), NeuroMuscular Research Center, Faculty of Sport and Health Sciences, University of Jyväskylä, Rautpohjankatu 8, 40014 Finland.

This file includes:

- Supplementary Figure 1
- Supplementary Figure 2
- Supplementary Figure 3
- Supplementary Figure 4
- Supplementary Figure 5
- Supplementary Figure 6

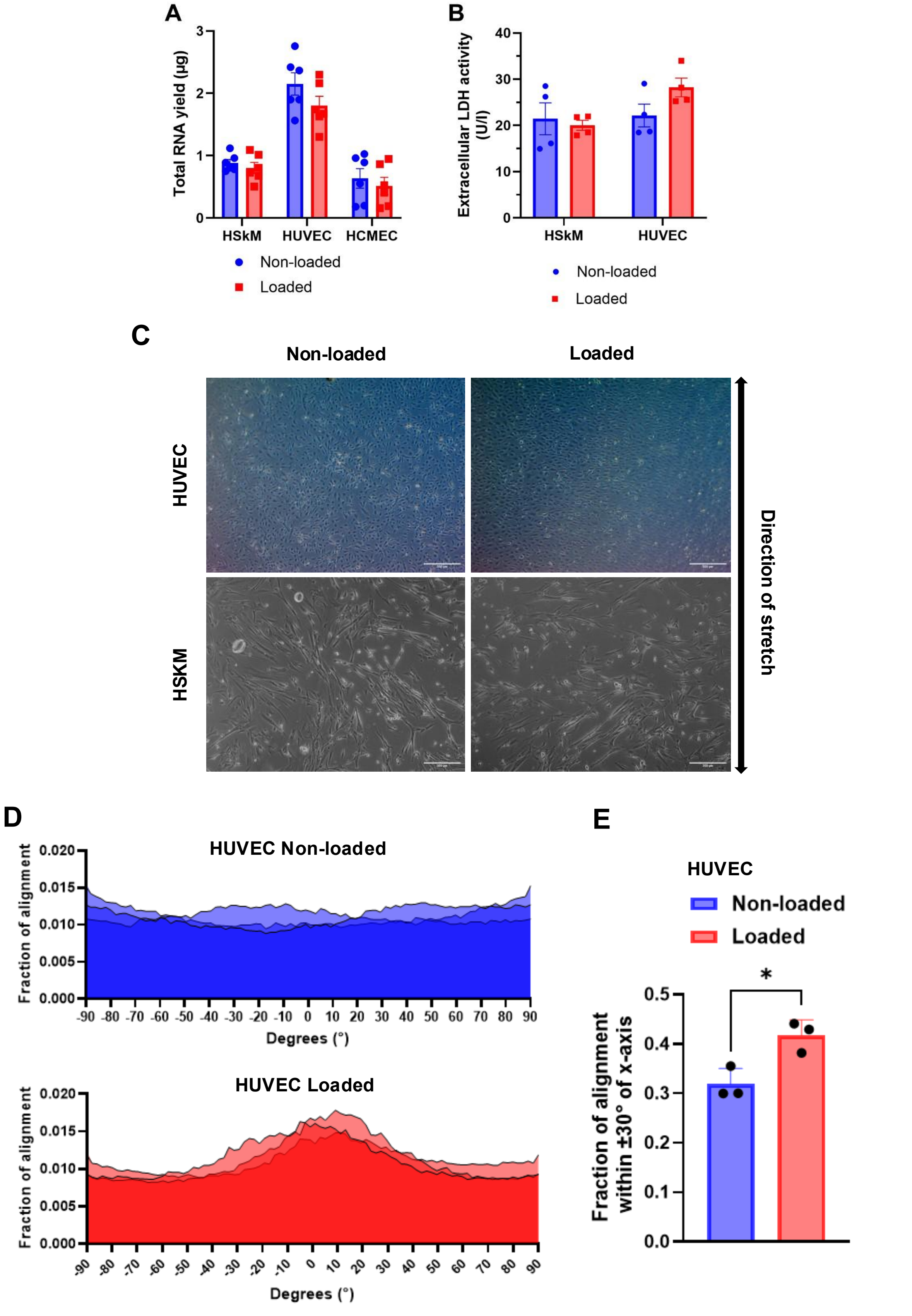

Supplementary Figure 1. Effects of mechanical loading on cell integrity, cell, membrane damage, cell viability and orientation of endothelial cells **A)** Total RNA yield was similar between

the non-loaded and loaded cells in all cell types, indicating that no significant detachment of cells occurred (n = 6). **B)** Mechanical loading did not significantly affect the extracellular lactate dehydrogenase (LDH) activity, a marker of cell membrane damage and cell death (n = 4). LDH activity was not determined from the media of HCMECs because the media were not collected from those experiments. **C)** Representative light-microscope images of HUVEC and Human skeletal muscle (HSkM) cells before and after mechanical loading. **D)** Histograms showing the orientation of HUVEC cells in non-loaded and loaded conditions (n=3, depicted in different shades) around the x-axis (perpendicular to the direction of stretch). **E)** Difference in the fraction of cells oriented perpendicular to the direction of stretch (±30º around the x-axis) between conditions. $^{*}p < 0.05$ (Student's t-test).

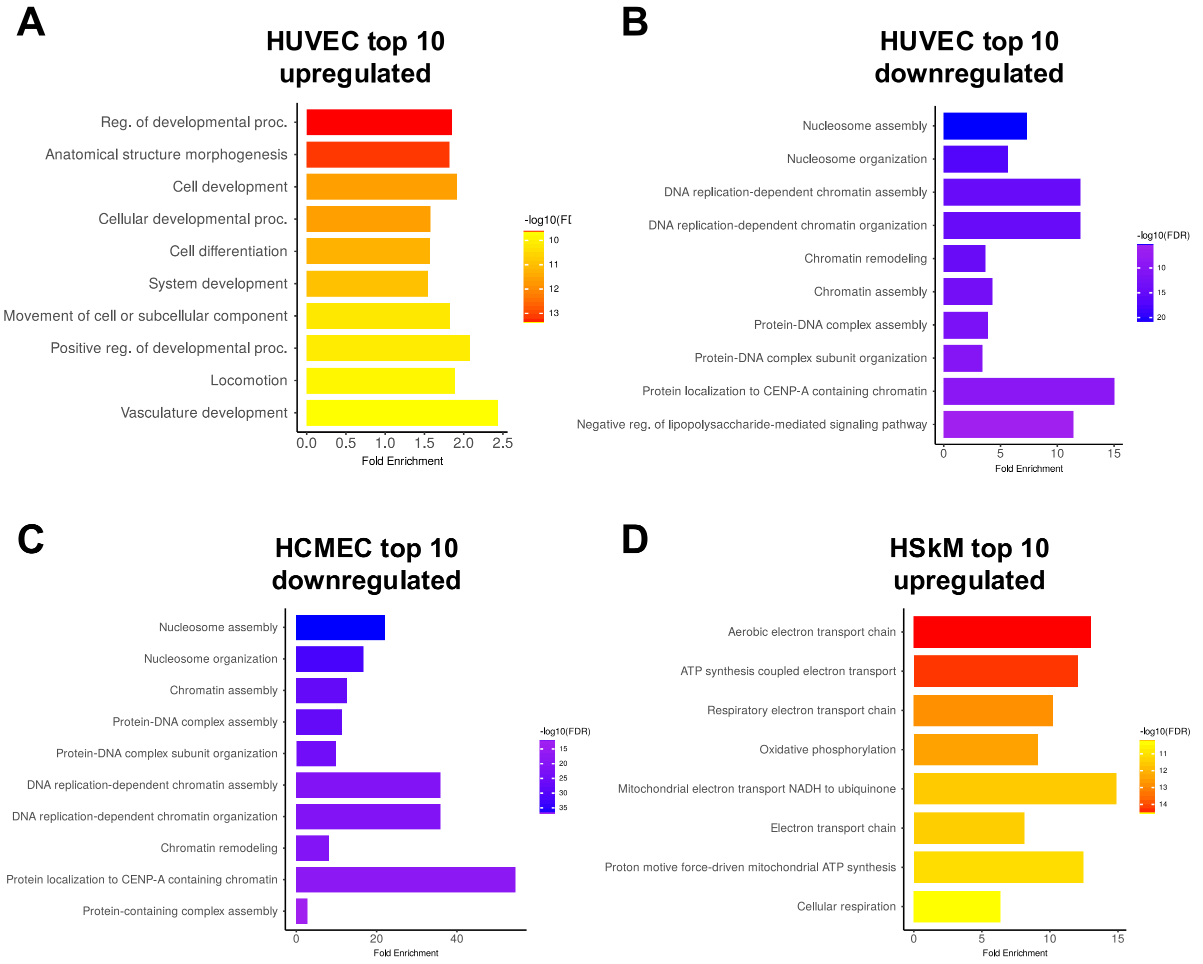

Supplementary Figure 2. Gene set enrichment of GO:BP terms from overrepresentation analysis with ShinyGO web-application. Analysis was done by comparing significantly upregulated or downregulated genes to background (all expressed genes in samples after filtering) **A)** HUVEC top 10 enriched pathways from upregulated genes. **B)** HUVEC top 10 enriched pathways from downregulated genes **C)** HCMEC top 10 enriched pathways from downregulated genes. **D)** HSkM top 10 enriched pathways from upregulated genes. No significantly enriched (FDR ≤ 0.05) GO:BP pathways were found from upregulated HCMEC genes or downregulated HSkM genes. Results were visualized by the ShinyGO web-application

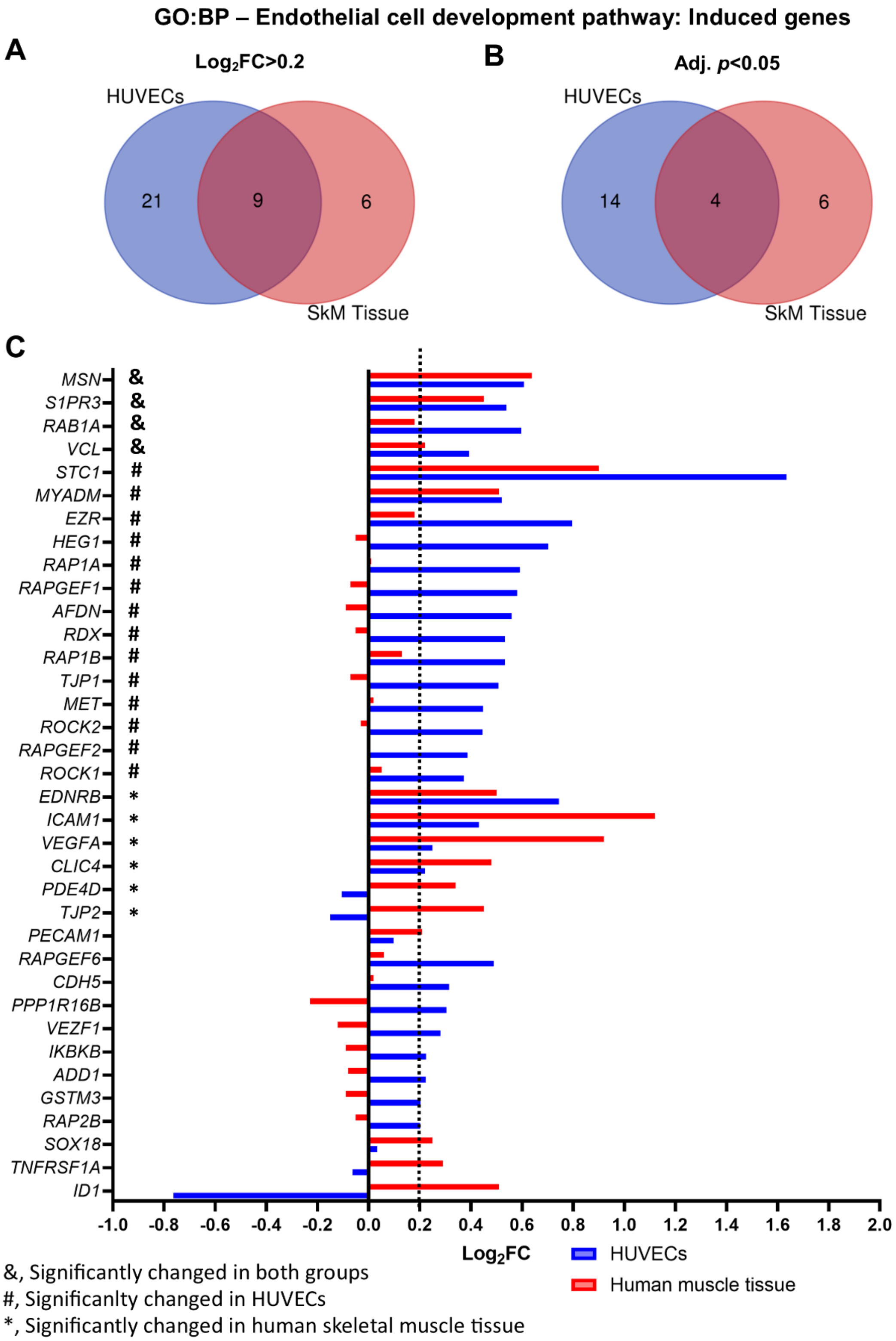

Supplementary Figure 3. Induction of genes of the *Endothelial cell development* pathway (GO:0001885) in response to mechanical loading in HUVECs and acute aerobic exercise in human skeletal muscle (HSkM) tissue. **A)** A Venn diagram of the induced genes. **B)** A Venn diagram of significantly (adj. p<0.05) upregulated genes. **C)** Graphical presentation of mean fold changes of the genes (out of 72 genes of the pathway) with $\log_2$FC>0.2 in HUVECs or HSkM tissue. The gene expression changes in response to acute aerobic exercise were checked from MetaMEx (https://www.metamex.eu/app/metamex, health status: healthy). The Venn diagrams were prepared with a web application (https://bioinformatics.psb.ugent.be/webtools/Venn/).

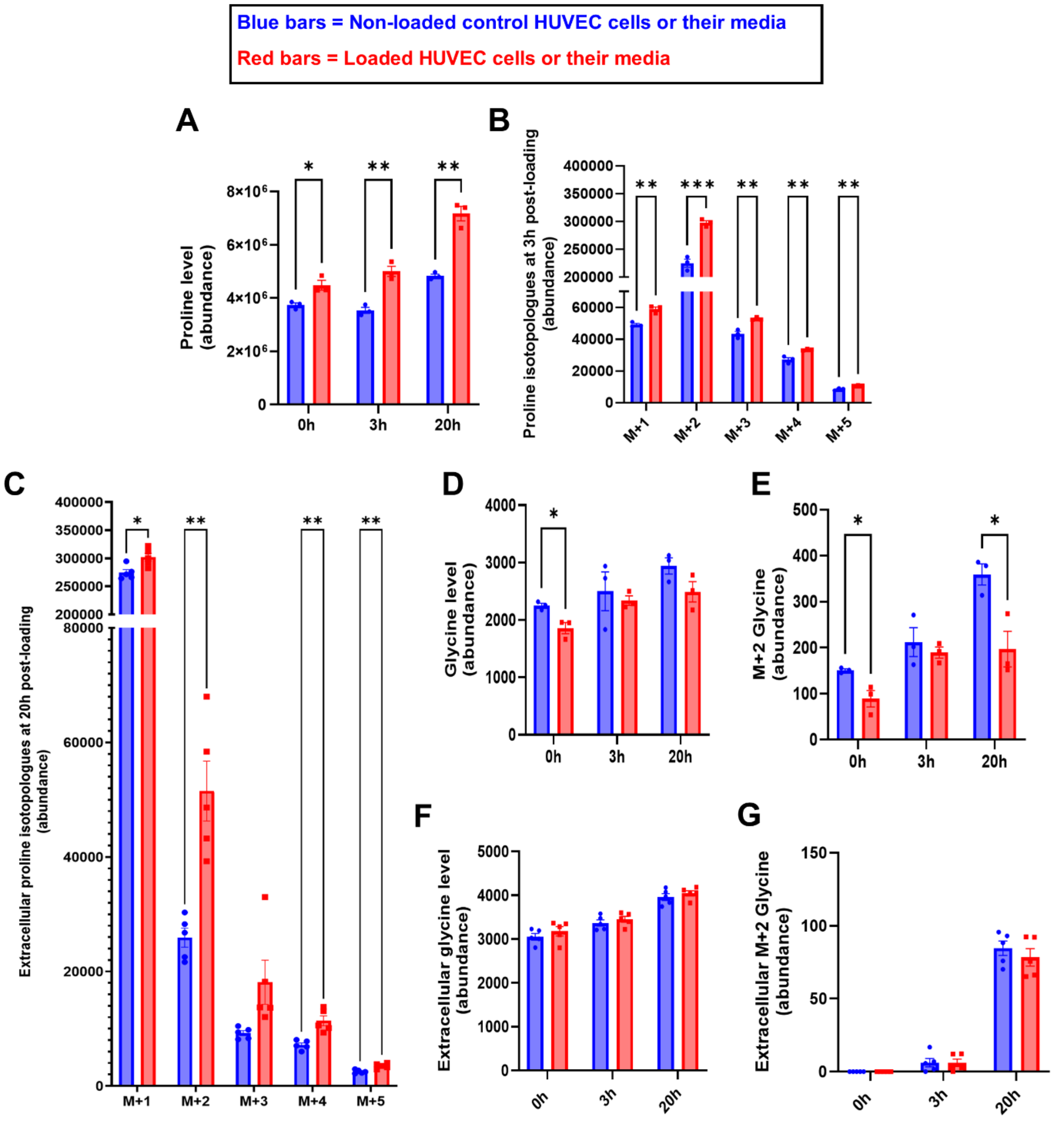

Supplementary Figure 4. ^{13}C-(U)-glucose-derived carbon incorporation into alanine, proline, and glycine in non-loaded (blue bars) and mechanically loaded (red bars) HUVECs at different time points after 5h mechanical loading.

A) Intracellular proline level (unlabeled + labeled).
B) Intracellular proline M+1-5 isotopologues at 3h post-loading.
C) Extracellular proline M+1-5 isotopologues at 20h post-loading.
D) Intracellular glycine level (unlabeled+labeled) at different time points.
E) Intracellular labeled (M+2) glycine level at different time points.
F) Extracellular glycine level (unlabeled+labeled) at different time points.
G) Extracellular labeled (M+2) glycine level at different time points.
Individual values are plotted, and data are presented as mean ± SEM.
*$P < 0.05$, **$P < 0.01$, ***$P < 0.001$* (Student's t-test).

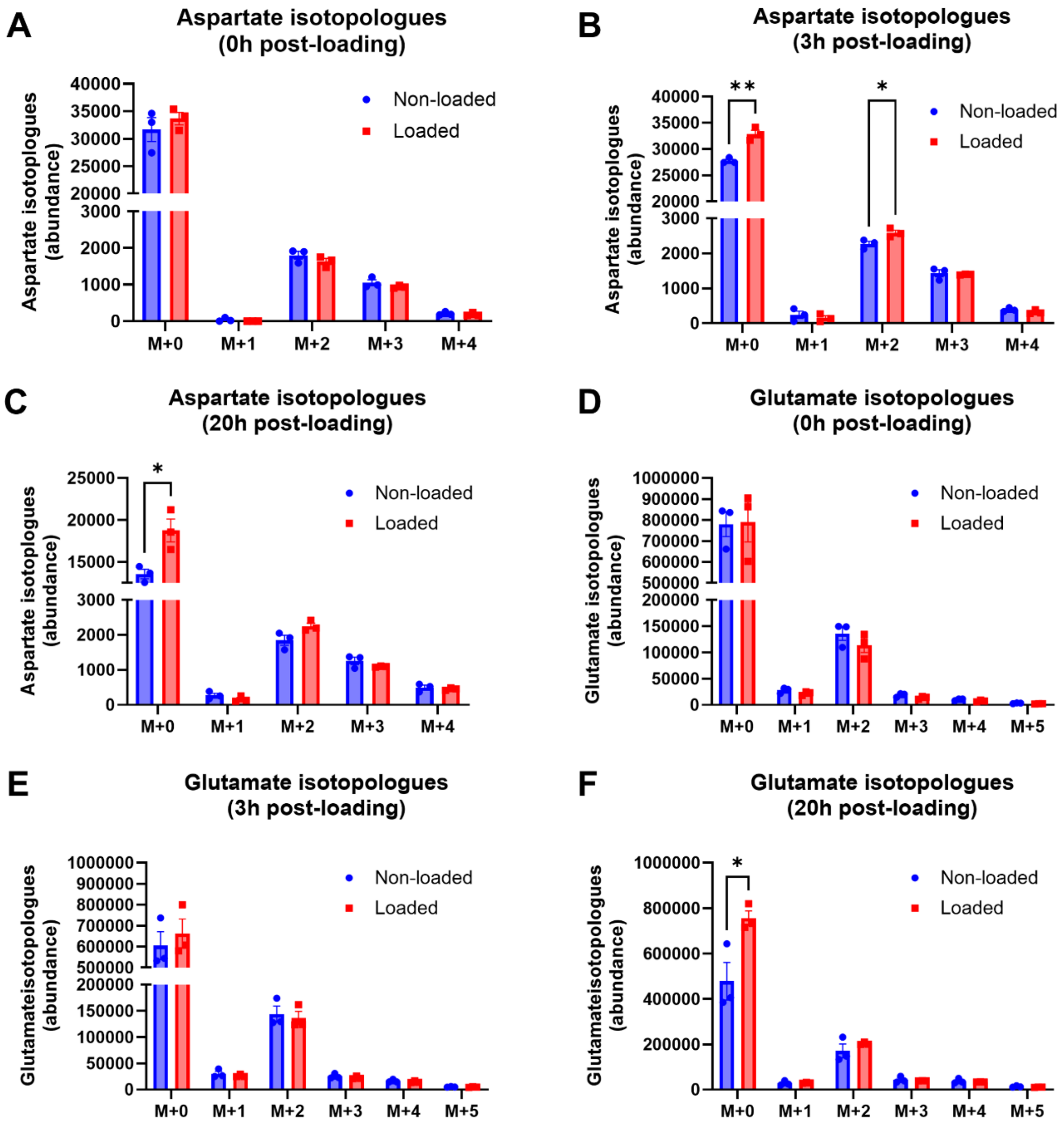

Supplementary Figure 5. Labeling patterns of intracellular aspartate and glutamate in non-loaded control and mechanically loaded (5h loading) HUVECs fed with ^{13}C-(U)-glucose.

A) Aspartate isotopologues at 0h post-loading.
B) Aspartate isotopologues at 3h post-loading.
C) Aspartate isotopologues at 20h post-loading
D) Glutamate isotopologues at 0h post-loading.
E) Glutamate isotopologues at 3h post-loading.
F) Glutamate isotopologues at 20h post-loading.
Individual values are plotted, and data are presented as mean ± SEM.
$*P < 0.05$, $**P < 0.01$(Student's t-test).

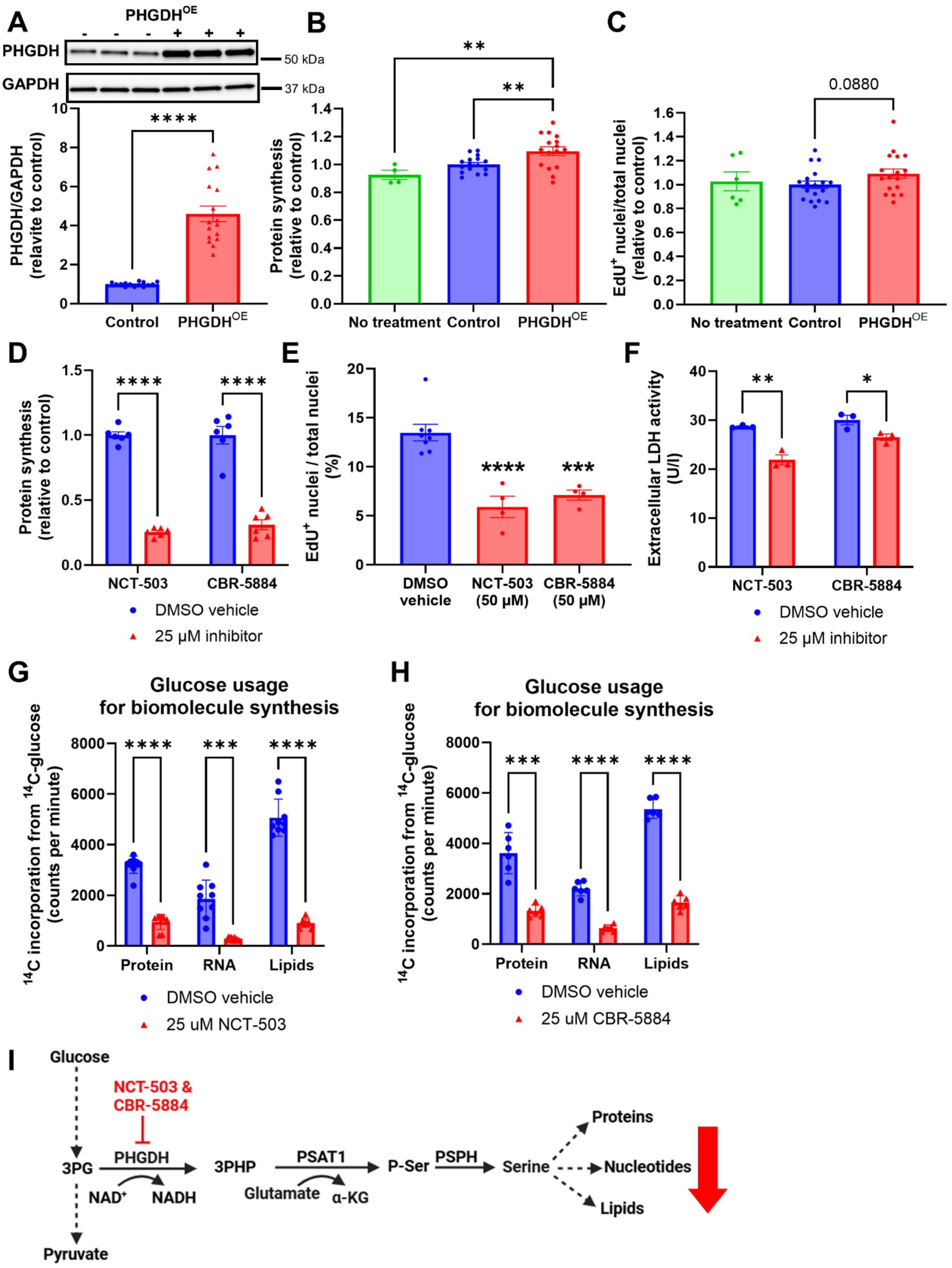

Supplementary Figure 6. Effects of the serine synthesis pathway enzyme phosphoglycerate dehydrogenase (PHGDH) overexpression and PHGDH inhibitors on anabolism of HUVECs.

A) PHGDH level in response to overexpression treatment (n = 16).

B) Protein synthesis (^{14}C-phenylalanine incorporation into proteins) in response to PHGDH overexpression treatment (n = 16, but in no treatment group n = 4).

C) Cell proliferation in response to PHGDH overexpression treatment (n = 24, but in the no-treatment group, n = 6).
D) protein synthesis (relative ^{14}C-valine incorporation into proteins) in response to PHGDH inhibitors NCT-503 and CBR-5884 (n = 6).
E) PHGDH inhibitors decreased the proliferation of HUVECs, as assessed by EdU analysis. The groups were statistically compared only to the DMSO vehicle control (n = 8 in the DMSO control group and n = 4 in the inhibitor groups).
F) PHGDH inhibitors did not induce cell death according to the lactate dehydrogenase (LDH) activity measured from conditioned media (i.e., increased LDH activity would indicate membrane damage and cell death) (n = 3). Individual values are plotted, and data are presented as mean ± SEM.
G) ^{14}C-U-glucose-derived carbon incorporation into proteins, RNA, and lipids in response to NCT-503 (n = 9).
H) ^{14}C-U-glucose-derived carbon incorporation into proteins, RNA, and lipids in response to CBR-5884 (n = 6).
I) Schematic presentation of the effects of PHGDH inhibitors.

Individual values are plotted, and data are presented as mean ± SEM. ****$p < 0.001$, **** $p <$ 0.0001. Student's t-test was used when two groups were compared and 1-ANOVA with Fisher's LSD test when more than two groups were compared (panels B, C & E).
